# Opportunistic Infections and Malignancies in a Patient With HIV/AIDS and a Critically Low CD4 Count of 1 Cell/μL

**DOI:** 10.7759/cureus.60129

**Published:** 2024-05-12

**Authors:** Sabrina Carpintieri, Elias Uyar, Yaroslav Buryk

**Affiliations:** 1 Internal Medicine, Ross University School of Medicine, Miami, USA; 2 Pulmonary and Critical Care, Jackson Memorial Hospital, Miami, USA

**Keywords:** opportunistic infections, hiv aids, cytomegalo virus (cmv), kaposi sarcoma, mycobacterium avium-complex

## Abstract

We present a 45-year-old African American male with a medical history of advanced-stage HIV/AIDS (CD4 count: 1 cell/μL) and poor adherence to highly active antiretroviral therapy (HAART), who presented with symptoms of diarrhea, weakness, and respiratory distress. Upon admission, duodenal and colonic biopsies revealed a diffuse histiocytic infiltrate consistent with *Mycobacterium avium* complex (MAC), and a cecal biopsy was positive for Kaposi sarcoma (KS). Further workup showed consolidation and a right pleural effusion on chest X-ray, suggesting a pneumonia infection. The patient's hypoglycemic state and lung consolidation raised concerns for sepsis, despite negative blood cultures for the first 24 hours. The patient was initiated on HAART and treated with azithromycin, rifabutin, and ethambutol for disseminated MAC. Despite the aggressive immunotherapy, the patient's condition did not improve, and he eventually expired. This case uniquely highlights the wide range of opportunistic infections and malignancies that can present in individuals with advanced-stage HIV/AIDS, underscoring the importance of early recognition and treatment. This susceptible demographic warrants further research due to the non-solidified prognosis of individuals with severe immunodeficiency.

## Introduction

A significant decrease in CD4+ T-cell count is a hallmark of HIV/AIDS. Individuals with advanced disease are at an increased risk of developing life-threatening opportunistic infections and malignancies. In patients with CD4 counts <50 cells/μL, *Mycobacterium avium* complex (MAC) is a common cause of disseminated mycobacterial infection [1,2].

HIV patients with immunosuppression and absence of antiretroviral therapy (ART) have a high incidence of developing disseminated MAC [2]. If untreated, this disease carries a very high mortality rate due to its ability to spread hematogeneously and involve multiple organs, most commonly the gastrointestinal tract, bone marrow, liver, and spleen.

Kaposi sarcoma (KS) is another AIDS-defining condition caused by human herpesvirus-8 (HHV-8) infection [3,4]. This endothelial malignancy has a preference for the gastrointestinal system and lungs but can also be seen in other visceral organs. The most common disease presentation is purple to pink lesions on the skin or mucocutaneous surfaces. While cutaneous lesions are the most common presentation, visceral involvement, particularly gastrointestinal KS, indicates a poorer prognosis.

We present a case of a 45-year-old African American male with HIV/AIDS and a critically low CD4 count of 1 cell/μL, who was admitted and later diagnosed with disseminated MAC colitis and visceral KS involving the colon and cecum [5-7]. This case emphasizes the devastating effects of an immunocompromised state and the importance of prompt recognition of these entities while maintaining a high clinical suspicion in severely immunocompromised patients presenting with nonspecific gastrointestinal symptoms. The diagnosis of disseminated MAC and visceral KS involvement are both relatively uncommon but carry severe prognosis, and pose an immense challenge for diagnosis and formulating an effective management plan.

## Case presentation

A 45-year-old African American male with a past medical history of HIV/AIDS presented to the emergency department with a two-week history of diarrhea and fatigue. He reported up to 10 episodes of watery, non-bloody diarrhea per day associated with diffuse abdominal cramping. He also complained of decreased appetite, generalized weakness, and night sweats.

At the time of presentation, his AIDS was poorly controlled with a CD4 count of 1 cell/μL determined through a laboratory test called flow cytometry and a high viral load of 3,230,000 copies/mL. Other pertinent history included non-adherence to HIV medications and prophylactic antibiotics, as well as polysubstance abuse, alcohol abuse, pancreatitis, hypothyroidism, anxiety/depression, and seizure disorder.

On admission to the emergency room, the patient's vital signs were recorded (Table 1). The concurrent laboratory investigations conducted upon admission are presented in Table 2.

**Table 1:** Patient vitals upon admission BPM, beats per minute; BRPM, breaths per minute.

Vital Signs Recorded	Recorded Values on Admission
Temperature	98 °F
Blood pressure	135/85 mmHg
Heart rate	122 BPM
Respiratory rate	20 BRPM
SpO2	97% on room air

**Table 2:** Initial laboratory investigation (H) indicates a high value outside the reference range; (L) indicates a low value outside the reference range; (!) indicates a critically low value. BUN, blood urea nitrogen; PT, prothrombin time.

Lab Results	Result	Reference Range	Units
CD4 count	1 (!)	500-1,600	cells/µL
HIV viral load	3,230,000	<20	copies/mL
White blood cell count	32.2 (H)	4.500-11,000	cells/µL
Hemoglobin	9.5 (L)	Men: 13.5-17.5	g/dL
Platelet count	18 (!)	150,000-400,000	mm^3^
Sodium	136 (L)	135-145	mmol/L
Potassium	4.5	3.5-5.0	mmol/L
Creatinine	3.30 (H)	0.7-1.3	mg/dL
BUN	60 (H)	7-20	mg/dL
PT	16.6 (H)	11.5-14.5	Seconds
INR	1.33	0.8-1.2	Ratio

The initial physical examination revealed cachexia with temporal wasting. The abdominal examination showed normoactive bowel sounds and diffuse tenderness to palpation without guarding or rebound. The remainder of the exam was notable for oral thrush and no other significant findings.

A series of diagnostic studies were performed to evaluate his condition. The tests revealed stool studies positive for Candida albicans (see Table 3). An esophagogastroduodenoscopy (EGD) revealed esophageal candidiasis. A CT scan of the abdomen and pelvis showed no evidence of hepatic or biliary pathology. A colonoscopy was performed, which revealed flat nodular mucosa in the cecum and nodular mucosa throughout the examined portion of the abdomen. Biopsies were taken during the colonoscopy, and the pathology results were significant. The duodenal biopsy showed diffuse histiocytic infiltrate of the lamina propria in the duodenal mucosa, consistent with MAC (see Table 3). The gastric biopsy revealed mild chronic gastritis, with no evidence of Helicobacter pylori seen on the H&E slides. The biopsy from a nodule at the gastroesophageal junction demonstrated squamocolumnar mucosa with erosion and rare epithelial cells with viral cytopathic changes consistent with CMV. The cecal biopsy was diagnostic of Kaposi's sarcoma (KS), while the colon biopsy also showed diffuse histiocytic infiltrate of the lamina propria, indicative of MAC (see Table 3).

**Table 3:** Biopsy/pathology study results MAC: *Mycobacterium avium* complex.

Biopsy/Pathology Study	Result
Duodenal biopsy	Diffuse histiocytic infiltrate consistent with MAC
Colon biopsy	Diffuse histiocytic infiltrate consistent with MAC
Cecal biopsy	Positive for Kaposi sarcoma

**Figure 1:**
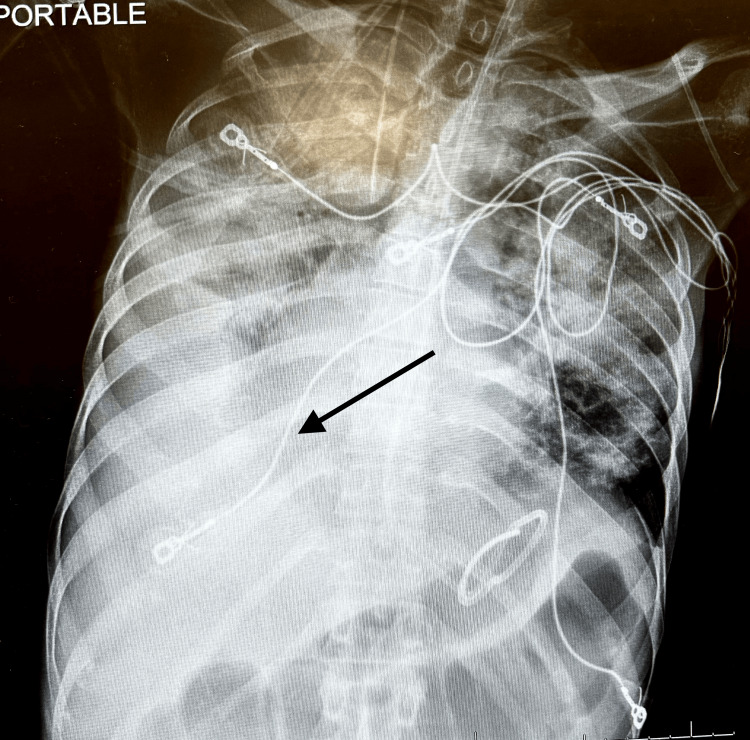
Chest X-ray The image displays a chest X-ray, and the arrow indicates a right-sided pleural effusion.

A chest X-ray (CXR) was obtained, which revealed a new right pleural effusion compared to his CXR on admission and a right lower lobe pneumonia (Figure 1). 

**Figure 2:**
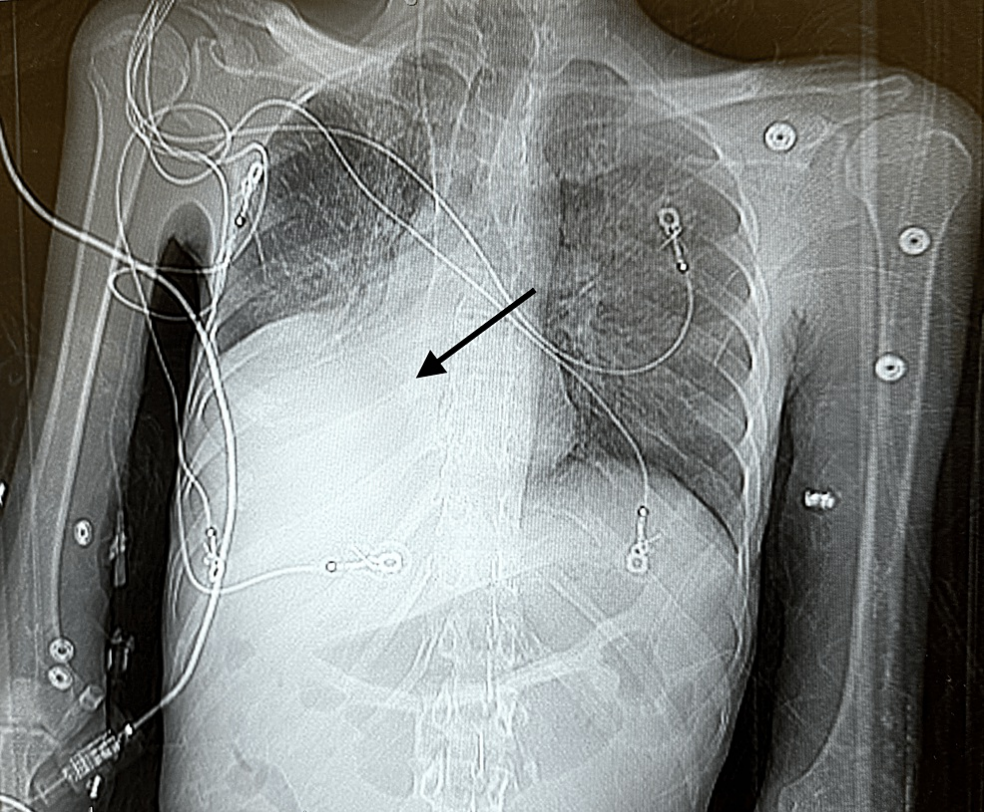
Chest CT without contrast The image displays a chest CT, and the arrow indicates a right lower lobe consolidation and a right-sided pleural effusion.

A CT chest confirmed pleural effusion with dense right lower lobe consolidation (Figure 2).

**Figure 3:**
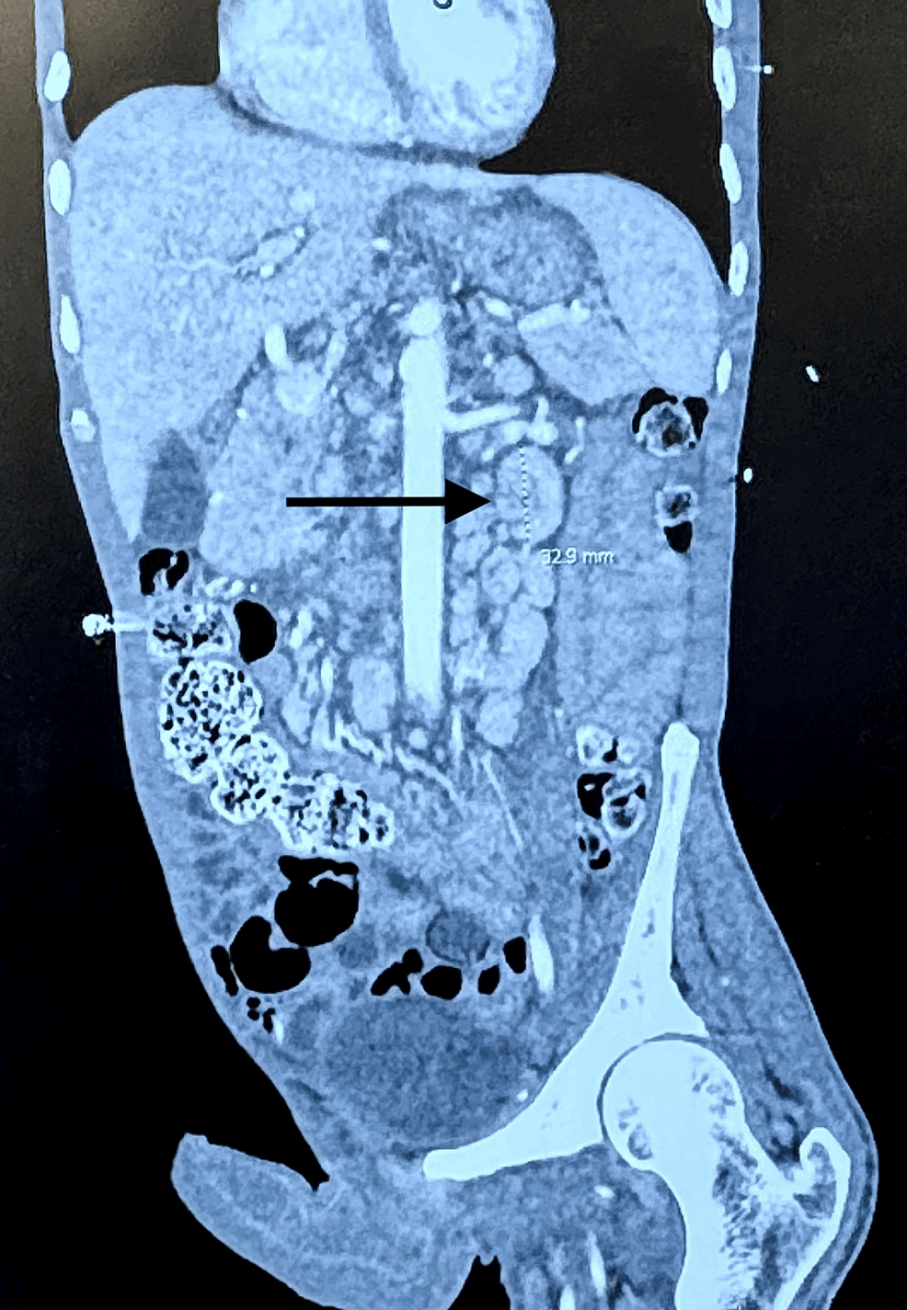
CT Abdomen sagittal cut The image displays an abdominal CT, and the arrow indicates retrocaval lymphadenopathy.

**Figure 4:**
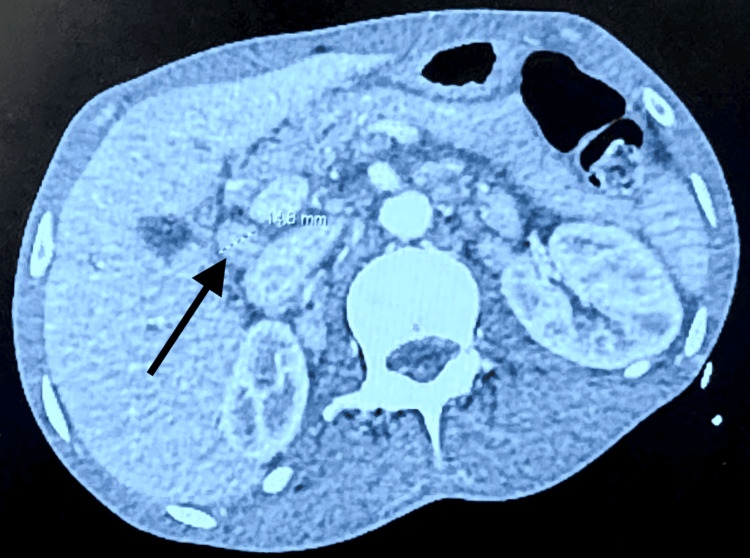
CT abdomen transverse cut The image displays an abdominal CT, and the arrow indicates portal caval lymphadenopathy.

The abdominal CT showed mild interval worsening of abdominal and retroperitoneal lymphadenopathy. Portal caval lymph nodes measured 1.8 cm, increased from 1.5 cm previously. Retrocaval lymph nodes measured 1.3 cm, up from 1.1 cm previously. Left para-aortic lymph nodes measured 2.1 cm, increased from 1.9 cm previously (see Figures 3, 4). 

The patient's repeated measurements of hypoglycemia, <10, despite being on D10 fluids, and lung consolidation raised suspicion for sepsis. The patient's HIV viral load was markedly elevated at 3,230,000 copies/mL. Stool acid-fast bacilli (AFB) testing was positive, likely secondary to disseminated MAC infection (see Table 4).

**Table 4:** Microbiologic study results

Microbiologic Study	Result
Stool studies	Positive for *Candida albicans*
Stool acid-fast bacilli (AFB) test	Positive, likely due to disseminated *Mycobacterium avium* complex (MAC) infection
Blood cultures	Negative

Highly active antiretroviral therapy (HAART) consisting of dolutegravir and emtricitabine/tenofovir was initiated, while treatment for disseminated MAC with azithromycin, rifabutin, and ethambutol continued. The critical care team managed the patient's comorbidities and complications including seizures, hypotension, pulmonary edema, esophageal candidiasis, transaminitis, diarrhea, MAC colitis, anxiety, depression, malnutrition, and concerns for refeeding syndrome and sepsis.

The patient was started on intravenous fluids, electrolyte repletion, and empiric antibiotic, antifungal, and antimotility therapy. However, his condition deteriorated with persistent diarrhea, development of hypoglycemia, and worsening respiratory distress. Due to low oral intake and refusal of the nasogastric (NG) tube, the patient experienced persistent weakness.

Due to respiratory distress, the patient was placed on bilevel positive airway pressure (BiPAP) with an inspiratory positive airway pressure (IPAP) of 20 cmH2O, an expiratory positive airway pressure (EPAP) of 5 cmH2O, and a respiratory rate of 7 breaths per minute. He required norepinephrine bitartrate to maintain normal mean arterial pressures (MAP). Laboratory studies revealed mild transaminitis, but CT of the abdomen and pelvis showed no hepatic or biliary pathology. The patient was reported to have black stools with a significant decrease in hemoglobin levels. He received one unit of packed red blood cells, and his hemoglobin normalized post-transfusion. The patient continued to have electrolyte derangements requiring repletion. Despite aggressive supportive care, the patient's clinical status continued to decline, and he passed away.

## Discussion

This case illustrates the complexity of opportunistic infections and malignancies in a patient with advanced HIV/AIDS and severe immunodeficiency. The patient was placed at an extremely high risk for developing life-threatening complications due to his critically low CD4 count of 1 cell/μL.

Disseminated MAC is a common opportunistic infection seen in patients with AIDS when the CD4 count falls below 50 cells/μL [1]. The non-specific symptoms including fever, cough, weakness, malaise, and diarrhea can make early diagnosis challenging. In our patient, the diagnosis of disseminated MAC was confirmed through a positive stool AFB test and the presence of diffuse histiocytic infiltrate in the duodenal and colonic biopsies. Systemic disease caused by MAC commonly involves the duodenum and colon [6], with gastrointestinal symptoms often including abdominal pain, malabsorption, and diarrhea.

Significant challenges are faced when determining the management of disseminated MAC and visceral KS in the setting of advanced AIDS. The initiation of HAART plays a crucial role in restoring immune function while monitoring for immune reconstitution inflammatory syndrome (IRIS). It also is known to reduce the incidence and improve the prognosis of KS [8]. The standard treatment for disseminated MAC is a three-drug regimen of ethambutol, rifabutin, and clarithromycin [9]. However, as seen in this patient, despite the initiation of appropriate medications, the prognosis remains poor in individuals with severe immunosuppression. The emphasis of this case is on recognizing the importance of considering multiple opportunistic infections and malignancies in patients with advanced HIV/AIDS with profound immunosuppression. The presence of CMV in the gastric biopsy and the high suspicion of sepsis based on hypoglycemia and lung consolidation underscore the need for a thorough diagnostic workup and a high index of suspicion for atypical presentations.

Managing such a case of profoundly advanced HIV/AIDS poses immense challenges, with three critical aspects requiring emphasis. Initially, the patient had a history of poor adherence to antiretroviral therapy (ART), which, combined with surrounding social determinants of health, acted as barriers preventing maintained viral suppression. Factors such as poverty, unstable housing, lack of transportation, food insecurity, and stigma can severely impair an individual's ability to consistently access healthcare and adhere to complex treatment regimens. Without a supportive environment to address these social determinants, even well-intentioned patients can struggle to maintain viral suppression. Most patients adhering to HAART are able to maintain higher CD4 counts and avoid the profound immunodeficiency seen here. Furthermore, in such advanced cases, IRIS poses a major risk if ART is initiated, with a paradoxical worsening of symptoms during the first few weeks as the recovering immune system mounts an exaggerated inflammatory response that can be life-threatening without prompt recognition and management.

Additionally, this patient population is also susceptible to other complications of advanced HIV like adrenal insufficiency due to CMV destruction of the adrenal glands, severe electrolyte disturbances, venous thromboembolism including pulmonary embolism, and overwhelming sepsis from common bacterial pathogens. A coordinated multidisciplinary approach is crucial to simultaneously address the underlying opportunistic infections, malignancies, and potential immune reconstitution phenomena while carefully monitoring for and preemptively treating these other dreaded complications.

The main limitations of this case report include the lack of long-term follow-up data and the absence of information regarding the patient's adherence to HAART and antimycobacterial therapy. Additionally, the specific treatment and response of visceral KS were not discussed in detail.

## Conclusions

This case highlights the considerable number of opportunistic infections and malignancies that can occur in patients with advanced HIV/AIDS. Improvement of outcomes is dependent on prompt recognition, early diagnosis, and aggressive management. However, the prognosis remains guarded due to IRIS and hospital-related complications in patients with severe immunosuppression, as demonstrated by the patient's eventual demise despite aggressive treatment. Future research should focus on developing new approaches for the prevention and management of opportunistic infections and malignancies in this vulnerable population.

## References

[1] Akram SM, Attia FN (2020). Mycobacterium avium intracellulare. StatPearls [Internet].

[2] (2019). Disseminated Mycobacterium avium complex disease. https://clinicalinfo.hiv.gov/en/guidelines/hiv-clinical-guidelines-adult-and-adolescent-opportunistic-infections/disseminated.

[3] Bishop BN, Lynch DT (2021). Kaposi sarcoma. StatPearls [Internet].

[4] Russo I, Marino D, Cozzolino C (2024). Kaposi’s sarcoma: evaluation of clinical features, treatment outcomes, and prognosis in a single-center retrospective case series. Cancers (Basel).

[5] Schneider JW, Dittmer DP (2017). Diagnosis and treatment of Kaposi sarcoma. Am J Clin Dermatol.

[6] Horsburgh CR Jr (179). The pathophysiology of disseminated Mycobacterium avium complex disease in AIDS. J Infect Dis.

[7] (2023). Kaposi sarcoma. https://www.hopkinsmedicine.org/health/conditions-and-diseases/sarcoma/kaposi-sarcoma.

[8] Martinez V, Caumes E, Gambotti L (2006). Remission from Kaposi's sarcoma on HAART is associated with suppression of HIV replication and is independent of protease inhibitor therapy. Br J Cancer.

[9] Benson CA (1994). Treatment of disseminated disease due to the Mycobacterium avium complex in patients with AIDS. Clin Infect Dis.

